# Transcriptome analysis of microRNAs, circRNAs, and mRNAs in the dorsal root ganglia of paclitaxel-induced mice with neuropathic pain

**DOI:** 10.3389/fnmol.2022.990260

**Published:** 2022-08-31

**Authors:** Qingxiang Mao, Lixia Tian, Jianxiong Wei, Xiaoqiong Zhou, Hong Cheng, Xuan Zhu, Xiang Li, Zihao Gao, Xi Zhang, Lingli Liang

**Affiliations:** ^1^Department of Anesthesiology, Daping Hospital, Army Medical University, Chongqing, China; ^2^Department of Physiology and Pathophysiology, School of Basic Medical Sciences, Xi’an Jiaotong University Health Science Center, Xi’an, China; ^3^Institute of Neuroscience, Translational Medicine Institute, Xi’an Jiaotong University Health Science Center, Xi’an, China

**Keywords:** paclitaxel, dorsal root ganglion, microRNA, circRNA, RNA sequencing

## Abstract

The microtubule-stabilizing drug paclitaxel (PTX) is a chemotherapeutic agent widely prescribed for the treatment of various tumor types. The main adverse effect of PTX-mediated therapy is chemotherapy-induced peripheral neuropathy (CIPN) and neuropathic pain, which are similar to the adverse effects associated with other chemotherapeutic agents. Dorsal root ganglia (DRG) contain primary sensory neurons; any damage to these neurons or their axons may lead to neuropathic pain. To gain molecular and neurobiological insights into the peripheral sensory system under conditions of PTX-induced neuropathic pain, we used transcriptomic analysis to profile mRNA and non-coding RNA expression in the DRGs of adult male C57BL/6 mice treated using PTX. RNA sequencing and in-depth gene expression analysis were used to analyze the expression levels of 67,228 genes. We identified 372 differentially expressed genes (DEGs) in the DRGs of vehicle- and PTX-treated mice. Among the 372 DEGs, there were 8 mRNAs, 3 long non-coding RNAs (lncRNAs), 16 circular RNAs (circRNAs), and 345 microRNAs (miRNAs). Moreover, the changes in the expression levels of several miRNAs and circRNAs induced by PTX have been confirmed using the quantitative polymerase chain reaction method. In addition, we compared the expression levels of differentially expressed miRNAs and mRNA in the DRGs of mice with PTX-induced neuropathic pain against those evaluated in other models of neuropathic pain induced by other chemotherapeutic agents, nerve injury, or diabetes. There are dozens of shared differentially expressed miRNAs between PTX and diabetes, but only a few shared miRNAs between PTX and nerve injury. Meanwhile, there is no shared differentially expressed mRNA between PTX and nerve injury. In conclusion, herein, we show that treatment with PTX induced numerous changes in miRNA expression in DRGs. Comparison with other neuropathic pain models indicates that DEGs in DRGs vary greatly among different models of neuropathic pain.

## Introduction

Neuropathic pain can be caused by a lesion in, or disease of, the somatosensory nervous system. Some of the most common conditions involving peripheral neuropathic pain, listed in the new classification criteria from the International Association for the Study of Pain (IASP), include trigeminal neuralgia, peripheral nerve injury, painful polyneuropathy, postherpetic neuralgia, and painful radiculopathy (Scholz et al., 2019). The toxicity of chemotherapeutic agents to the peripheral nervous system can also induce peripheral neuropathy and neuropathic pain (Liang et al., 2020a; Starobova et al., 2020). Most patients with neuropathic pain report an ongoing or intermittent spontaneous pain and pain evoked by light touch or other stimuli (Finnerup et al., 2021). Ectopic activity in injured nerves or dorsal root ganglia (DRG), and peripheral and central sensitization, underlie spontaneous and evoked pain in different neuropathic pain conditions (Finnerup et al., 2021). Multiple mechanisms, including alteration in ion channel activity, activation of immune cells, glial-derived mediators, and epigenetic regulation in the peripheral sensory neurons, contribute to the neurogenesis of neuropathic pain (Liang et al., 2015; Finnerup et al., 2021). However, the various neuropathic pain conditions involve different underlying mechanisms.

Paclitaxel (PTX) is a well-known chemotherapeutic agent with a unique mechanism of action. PTX binds to microtubules in the cytoskeleton and enhances tubulin polymerization, resulting in cellular apoptosis (Jordan and Wilson, 2004). Treatment using PTX can also lead to peripheral neuropathy, which is similar to the adverse effects exerted by other chemotherapeutic agents (Mao et al., 2019; Liang et al., 2020a; Starobova et al., 2020; Kang et al., 2021). Previously, we have reported on an epigenetic regulation mechanism in paclitaxel-induced mouse model of neuropathic pain. In that study, we described that DNA methyltransferase DNMT3a triggers downregulation of *K2p 1.1* expression in the DRG, which contributes to paclitaxel-induced neuropathic pain (Mao et al., 2019). To elucidate more potential mechanisms driving PTX-induced neuropathic pain, herein we used high-throughput RNA sequencing (RNA-Seq) and transcriptomic analysis to evaluate gene expression changes in the DRGs of PTX-treated mice. Previous studies have utilized transcriptomic analysis conducted using high-throughput RNA-Seq or microRNA arrays to profile gene-expression changes in the DRGs of mice with neuropathic pain induced by nerve injury (Wu et al., 2016), diabetes (Zhang H. H. et al., 2020), or other chemotherapeutic agents (Starobova et al., 2020). Hence, we further compared PTX-induced differentially expressed miRNAs, circRNAs, and mRNAs with those described in other neuropathic pain models. RNA-Seq and in-depth gene expression analysis revealed that 345 microRNAs, 6 mRNAs, 3 lncRNAs, and 16 circRNAs were differentially expressed in response to treatment utilizing PTX. Nevertheless, there are only a few shared differentially expressed genes in these neuropathic pain models. Gene expression in the DRG varies depending on the type of insult to these neurons or their axons. Although transcriptomic gene expression profiling enhances our understanding of pain mechanisms under various neuropathic pain conditions, further investigations are needed to validate the potential molecular targets to develop individualized treatment strategies for patients with neuropathic pain.

## Materials and methods

### Animals

Adult male C57BL/6 mice (8–9 weeks old) were used in this study. Mice were housed (up to five per cage) at 25°C and maintained at a standard 12-h light/dark cycle, with water and food available *ad libitum*. The animal housing facility and all procedures involving animals were approved by the Institutional Animal Ethics Committee of the Xi’an Jiaotong University Health Science Center, and were conducted in accord with the ethical guidelines put forth by the International Association for the Study of Pain. All efforts were made to minimize the suffering of laboratory animals and to reduce the number of animals used in the study. All investigators performing experiments were blinded to the group designation of the mice.

### Treatment using paclitaxel

Paclitaxel was prepared and administered as described previously (Liang et al., 2020a). Briefly, to prepare 1 mL of PTX stock solution at 6 mg/mL concentration, PTX (6 mg; MedChemExpress, Monmouth Junction, NJ, United States) was dissolved in a 1:1 solution of Cremophor EL (500 μL; Sigma-Aldrich, St. Louis, MO, United States) and ethanol (500 μL). Prior to treatment, the PTX stock solution was diluted with 0.9% sterile sodium chloride (NaCl) to a final concentration of 0.4 mg/mL. Each mouse was injected with PTX (4 mg/kg) intraperitoneally (i.p.) every other day for a total of six times. The control mice received vehicle solution (Cremophor EL: ethanol, 1:1, diluted using 0.9% sterile NaCl solution) using the same schedule as that used for PTX mice.

### Behavioral tests

All mice were habituated to the testing environment for 3 days prior to baseline testing. Von Frey filaments (0.07 and 0.40 g; Stoelting Co., Wood Dale, IL, United States) were used to evoke mechanical allodynia and hyperalgesia as described previously (Liang et al., 2020a,b). Briefly, each mouse was placed onto an elevated mesh screen within a Plexiglas chamber and was allowed an accommodation period until exploration and grooming behavior ceased. Each calibrated von Frey filament was applied to the left or right hind paw for approximately 1 s, and each trial was repeated 10 times at intervals of 3 min between each application. A positive response occurred when the mouse showed a strong paw withdrawal response to the von Frey filament. Both positive and negative responses were recorded in each trial and the number of positive responses in the 10 times was expressed as percentage of paw withdrawal frequency (PWF) [(number of paw withdrawals/10 times) × 100 = % PWF].

Evoked thermal hyperalgesia to noxious heat was assessed using a Model 37,370 Analgesic Meter (UGO Basile, Comerio, Italy) (Liang et al., 2020b,c). Briefly, each mouse was placed onto a glass plate within a Plexiglas chamber, and radiant heat was applied from below to the middle of the plantar surface of each hind paw until paw withdrawal occurred. When the mouse lifted its foot, the light beam was turned off automatically. The length of time from the starting of the light beam and the foot lift was defined as paw withdrawal latency (PWL). The cut-off time was 20 s to avoid tissue damage. Each trial was repeated five times for one paw at 5-min intervals.

### RNA-seq, bioinformatics, and pathway analysis

Mice were euthanized and bilateral lumbar 3 (L3) to L5 DRGs were harvested and flash frozen in liquid nitrogen, and then stored at −80°C. Bilateral L3-L5 DRGs obtained from 2 mice suffering from PTX or vehicle injection were pooled and used as one biological replicate. Six independent biological replicates collected from twelve mice in the vehicle and PTX groups (three biological replicates per group) were sent to BGI Genomics Inc. (Wuhan, China) described previously (Liang et al., 2020a). Briefly, total RNA from each sample was extracted and purified using the TRIzol method. The quality of RNA was estimated by an RNA Integrity Number (RIN) between 1 and 10 with 10 being the highest quality samples showing the least degradation. The RIN scores of six samples were shown in Supplementary Datasheet 1. The RIN score of each sample is over 6, indicating that they are of sufficient quality to produce a library for sequencing (Kukurba and Montgomery, 2015). Then, RNA sequencing was conducted on a BGISEQ500 platform (BGI Genomics). Ten samples were evaluated using multiplexing, sequencing, differential gene expression analysis, and transcriptomic expression analysis. Raw data were quality controlled using SOAPnuke software (BGI Genomics) (Cock et al., 2010) and clean reads were obtained after filtering out reads with ribosome RNA (rRNA) and other contaminations. Clean reads were mapped to *Mus musculus* genome sequence (version GCF_000001635.26_GRCm38.p6) from the National Center for Biotechnology Information (NCBI) using hierarchical indexing for spliced alignment of transcripts (HISAT; Center for Computational Biology, Johns Hopkins University, Maryland, MD, United States), and to *Mus musculus* gene sequence using Bowtie2 (Langmead and Salzberg, 2012), to quantify mRNAs and long non-coding RNAs. To quantify circRNAs, clean reads were mapped to known *M. musculus* circRNAs. To detect small RNAs, clean tags were mapped to an miRNA database and *M. musculus* genome sequence, and then quantified. Mapped reads for each gene were calculated to determine the expression levels for that gene. Differential expression analysis was performed via MA-plot software using the DEGseq method (Wang et al., 2010). All expression values were transformed into log_2_ values. Comparisons of gene expression levels between groups were conducted using Student’s *t*-test. Differentially expressed genes (DEGs) were designated as genes with adjusted *P*-values (*Q*-values) less than 0.05. Log_2_ fold changes (FC) in miRNA expression were either more or less than 1. For analysis of DEG mRNA, functional classifications and pathways were evaluated using Gene Ontology (GO) Elite and DAVID Bioinformatics Resources 6.7 Kyoto Encyclopedia of Genes and Genomes (KEGG) pathways. All these data can be extracted manually by utilizing Dr. Tom on-line analysis system provided by BGI Genomics Inc.

### Reverse transcription-polymerase chain reaction for detection of microRNA

Bilateral L3–L5 DRGs collected from each mouse were pooled to obtain a sufficient amount of RNA. Tissues were homogenized in, and total RNA was extracted using, Beyozol (Beyotime, Shanghai, China) according to the manufacturers’ protocol. For miRNA quantification, 200 ng total RNA was reverse-transcribed into cDNA using a stem-loop primer specific for each mature miRNA, and using an miRNA First Strand cDNA Synthesis kit (Stem-loop Method) (Sangon Biotech, Shanghai, China) according to manufacturer’s instructions. Quantitative PCR was performed on a Real-time Detection System (Agilent Mx3005P qPCR system, Santa Clara, CA, United States) using Hieff^®^ qPCR SYBR^®^ Green Master Mix (Yeasen Biotechnology, Shanghai, China), and forward and reverse primers specific for each miRNA. All the primers used in this procedure (Table 1) were designed and synthesized by Sangon Biotech (Shanghai, China). PCR reactions were performed as follows: initial 3-min incubation at 95°C, followed by 40 cycles at 95°C for 10 s (s), 60°C for 30 s, 72°C for 30 s, 95°C for 5 s, 65°C for 15 s, and 95°C for 5 s. PCR products were verified using agarose gel electrophoresis and analysis of dissociation curve. U6 small nuclear RNA (Rnu6) was used as endogenous control to normalize differences in miRNA levels of each sample. Quantification was performed by normalizing target gene cycle threshold (Ct) values to that of Rnu6 Ct. The ratio of relative miRNA level in each sample to average miRNA level in vehicle-group samples was calculated using the 2^–ΔΔ^ ^Ct^ method.

**TABLE 1:** The miRNA primers.

MiRNAs	Primers	Sequences (5′–3′)
mmu-miR-376b-3p	RT	GTCGTATCCAGTGCAGGGTCCGAGGTATTCGCACTGGATACGACAAGTGG
	Forward	AAGCGCCTATCATAGAGGAACA
	Reverse	CAGTGCAGGGTCCGAGGT
mmu-miR-29c-3p	RT	GTCGTATCCAGTGCAGGGTCCGAGGTATTCGCACTGGATACGACTAACCG
	Forward	AGCTGGACTAGCACCATTTGAAA
	Reverse	AGTGCAGGGTCCGAGGTATT
mmu-miR-532-5p	RT	GTCGTATCCAGTGCAGGGTCCGAGGTATTCGCACTGGATACGACACGGTC
	Forward	AACCTCCCATGCCTTGAGTG
	Reverse	CAGTGCAGGGTCCGAGGT
Rnu6 (*Mus musculus*)	RT	GTCGTATCCAGTGCAGGGTCCGAGGTATTCGCACTGGATACGACAAAAAT
	Forward	GAAGATTTAGCATGGCCCCTGC
	Reverse	CAGTGCAGGGTCCGAGGT

RT, reverse transcription.

### Reverse transcription-polymerase chain reaction for detection of circRNA

For quantification of circRNA expression levels, total RNA was reverse-transcribed using ThermoScript reverse transcriptase and Random Hexamer Primer (Invitrogen/Thermo Fisher Scientific). For quantitative RT-PCR (qRT-PCR), each sample was run in triplicate using 20 μl reaction volume comprised of 10 μl Hieff^®^ qPCR SYBR^®^ Green Master Mix, 20 ng cDNA, and 2 μL 250 nM forward and reverse primers. All the primers used in this procedure (Table 2) were designed and synthesized by Sangon Biotech (Shanghai, China). PCR reactions were run on an Agilent Mx3005P qPCR system using the following conditions: initial 3-min incubation at 95°C, 40 cycles at 95°C for 10 s, 60°C for 30 s, 72°C for 30 s, 95°C for 5 s, 65°C for 15 s, and 95°C for 5 s. Agarose gel electrophoresis and analysis of dissociation curve were used to verify PCR products. All data were normalized to expression levels of *Gapdh* used as internal control. The ratio of mRNA level in each sample to average mRNA level in samples from the vehicle treatment group was calculated using the 2^–ΔΔCT^ method.

**TABLE 2:** The primers for circRNAs and the internal control gene.

CircRNAs	Primers	Sequences (5′–3′)
mmu_circ_0009357	Forward	ACCACGAGAATGCGAAGGAACAAG
	Reverse	CCTCCTGTCATCCTCCTCATCTCC
mmu_circ_0013069	Forward	GGGGTGTAGAGGGCAAGGAGAG
	Reverse	TCTTGTTCTTCTTTCCTGCCTTCCC
mmu_circ_0006031	Forward	GTGGAGGTGAGGCAGGAGAGTC
	Reverse	ACCTGAGGTGTCCCGCTTCTTG
mmu_circ_0001817	Forward	ACCGGTCCTCCTCTATTCGG
	Reverse	CCAAACAAGCTCTCAAGGTCCA
mmu_circ_0011289	Forward	GGAGACTGTGCCTGTGGTTGTG
	Reverse	TCAGTCCTCTCAGCCTCCATATTCC
*Gapdh*	Forward	TCGGTGTGAACGGATTTGGC
	Reverse	TCCCATTCTCGGCCTTGACT

### Statistical analysis

For behavioral tests, 24 mice were randomly distributed into the vehicle and PTX groups (12 mice/group). Data obtained using behavioral tests are expressed as means ± error of mean (SEM), and were statistically analyzed using two-way repeated measure (RM) ANOVA followed by *post hoc* Tukey test (SigmaPlot 12.5, San Jose, CA, United States). Three biological repeats (two mice/repeat) were selected randomly from 10 mice in each group and examined using RNA-Seq. Three to six mice (one mouse/repeat) per group were examined using RT-PCR and were statistically analyzed using two-tailed unpaired *t*-test (SigmaPlot 12.5). *P*-values less than 0.05 were considered statistically significant.

## Results

### Paclitaxel induces hypersensitivity to pain

**FIGURE 1:**
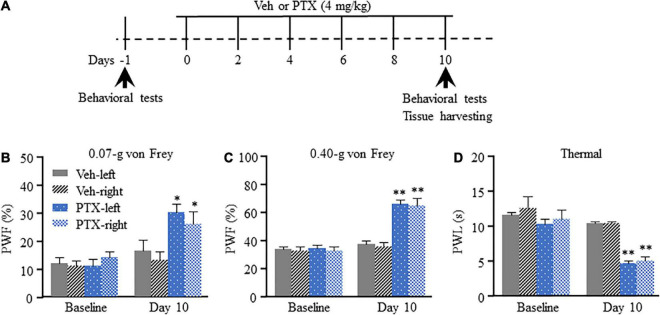
Mechanical allodynia and thermal hyperalgesia induced by paclitaxel (PTX) in mice. **(A)** A schematic of drug administrations and behavioral tests showed that mice were intraperitoneally injected with PTX (4 mg/kg) or vehicle solution (200 μl) every other day for a total of six times. **(B–D)** The paw withdrawal frequency (PWF) in response to a 0.07-g **(B)** and 0.40-g **(C)** von Frey filament, and the paw withdrawal latency (PWL), in response to thermal stimulation **(D)**, were assessed on day 10 after the first injection using PTX, and were compared with those of mice in the vehicle (Veh)-treated group. *n* = 12/group. Results are presented as mean ± SEM; *P < 0.05, ^**^P < 0.01, compared with vehicle group by two-way ANOVA followed by *post hoc* Tukey test.

We have previously shown that intraperitoneal injection of PTX induces pain hypersensitivity which peaks at approximately 10–14 days after the first injection (Mao et al., 2019). Therefore, day 10 was selected as time point for evaluation of PTX-induced transcriptomic changes in the DRG using RNA sequencing. To ensure that all mice treated using PTX showed pain hypersensitivity, paw withdrawal frequency (PWF) in response to mechanical stimulation and paw withdrawal latency (PWL) in response to thermal stimulation were assessed on day 10 following the first PTX injection and before the harvesting of DRGs (Figure 1A). In agreement with results obtained in our previous studies (Mao et al., 2019; Liang et al., 2020a), our present results show that treatment using PTX led to a dramatic increase in PWF response to 0.07- and 0.40-g von Frey filaments in both hind paws on day 10 compared with baseline response and that of the vehicle-treated group (*P* < 0.05 for 0.07-g von Frey filament; *P* < 0.01 for 0.40-g von Frey filament; Figures 1B,C). Similarly, treatment using PTX also induced thermal hyperalgesia in our mice, as shown by decreased PWL response in both hind paws compared with baseline response and that of the vehicle-treated group (*P* < 0.01; Figure 1D). Treatment with vehicle did not induce changes in either PWF or PWL of either hind paw compared to their respective baseline response levels (Figures 1B–D).

### Paclitaxel induces transcriptomic changes in coding and non-coding RNAs

**FIGURE 2:**
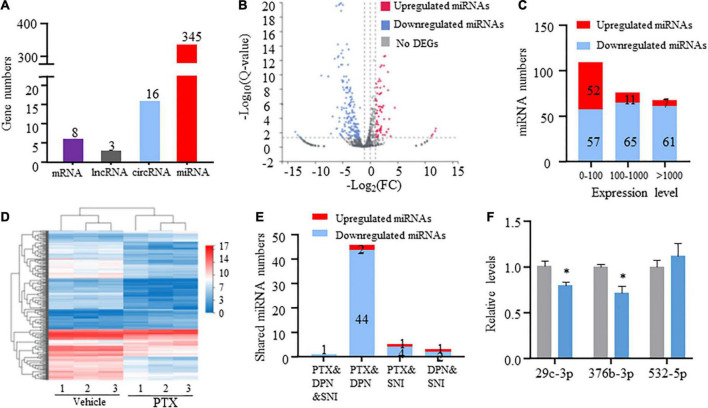
Transcriptome profiling of miRNAs in paclitaxel (PTX)-treated mice. **(A)** Numbers of differentially expressed genes (DEGs) were determined using transcriptome analysis. DEGs are genes having *Q*-values less than 0.05. **(B)** Volcano plot of differentially expressed miRNAs after PTX administration shows the most highly upregulated [log_2_(fold change, FC) > 1] or downregulated [log2(FC) < −1] genes. **(C)** Numbers of upregulated and downregulated miRNA showing low (0–100), medium (100–1,000), or high (more than 1,000) expression levels, based on respective expression level values in the vehicle group. **(D)** Hierarchical clustering analysis shows 253 differentially expressed miRNAs in vehicle and PTX groups. **(E)** Numbers of differentially expressed miRNA shared among PTX, diabetic peripheral neuropathy (DPN), and spared nerve injury (SNI) models. **(F)** Validation of PTX-induced downregulation of miR-376b-3p and miR-29c-3p expression as assessed using quantitative polymerase chain reaction. Results are presented as mean ± SEM of 3–6 independent experiments. *P < 0.05 compared with the vehicle group by two-tailed unpaired *t*-test.

RNA-Seq and in-depth gene expression analysis were used to explore transcriptomic changes in mRNA and non-coding RNA expression in PTX-induced DRGs. Our analyses identified a total of 67,228 genes including 22,899 coding mRNAs, 24677 lncRNAs, 16127 circRNAs, 2959 microRNAs, and 554 pseudoRNAs. DEGs were first extracted based on their *Q*-values of being less than 0.05. Our results indicate that 345 microRNAs were differentially expressed in response to treatment using PTX, while only 8 mRNAs, 3 long non-coding RNAs (lncRNAs), and 16 circRNAs were extracted as DEGs after induction using PTX (Figure 2A).

### Differentially expressed miRNAs induced by paclitaxel treatment

Next, we used threshold values log_2_FC > 1 or log_2_FC < −1 to identify a total of 253 differentially expressed miRNAs that comprised 70 upregulated and 183 downregulated miRNAs (Figures 2B,C and Supplementary Datasheet 2). Hierarchical clustering analysis revealed 253 differentially expressed miRNAs in the vehicle and PTX groups (Figure 2D). We also defined miRNA expression levels as low (0–100), medium (100–1,000), or high (more than 1,000) based on their respective levels in the vehicle group (Supplementary Datasheet 2). Our results indicate that nearly equal numbers of miRNAs were upregulated or downregulated in the low expression group. However, PTX induced a downregulation in the expression levels of most miRNAs in the medium and high expression groups (Figures 2B,C). These findings suggest that miRNA basal expression levels are an important factor in their expression changes after treatment using PTX.

Of the identified 253 differentially expressed miRNAs, the top 11 significantly upregulated and top 22 significantly downregulated miRNAs with medium or high expression levels are listed in Table 3. We then searched studies in PubMED (up to July 09, 2022) to investigate whether these miRNAs have been previously reported in pain-related conditions. The results of our search indicate that 10 miRNAs in the DRGs were reported to be involved in pain regulation induced by nerve injury, diabetes, or inflammation (Rau et al., 2010; Zhang et al., 2011; Wang et al., 2014; Sakai et al., 2017; Jia et al., 2018; Dai et al., 2019; Zhang and Wang, 2019; Xu et al., 2020; Zhang Z. et al., 2020; Li et al., 2021) (Table 3). However, changes in the expression levels of some of these miRNAs are opposite under different pain conditions. Because changes in miRNA expression profiles have been reported in spared nerve injury (SNI)-induced neuropathic pain (Dai et al., 2019) and diabetic peripheral neuropathy (DPN) (Bali et al., 2021), we then compared PTX-induced DEGs in the DRGs of our mice with those in SNI-induced or diabetic neuropathic pain models. Our results indicate that only one miRNA, mmu-miR-532-5p, showed consistent changes in expression among these neuropathic pain models (Table 4). PTX and DPN shared the highest number of DEGs; among 46 DEGs in total, only 2 were upregulated and the remaining 44 were downregulated. PTX and nerve injury shared five differentially expressed miRNAs, while DPN and nerve injury shared three DEGs (Figure 2E and Supplementary Datasheet 3). The differentially expressed miRNAs shared among these models may contribute to neuropathic pain irrespective of causative origin. We then used qPCR to validate the expression levels of mmu-miR-29c-3p, mmu-miR-376b-3p, and mmu-miR-532-5p shown in Tables 3, 4, respectively. Our results indicate that PTX induced a downregulation in the expression levels of mmu-miR-376b-3p and mmu-miR-29c-3p (Figure 2F), which agreed with our results obtained using RNA-Seq. Nonetheless, qPCR indicated that the expression level of mmu-miR-532-5p was not modified by treatment using PTX (Figure 2F).

**TABLE 3:** The top upregulated or downregulated miRNAs induced by paclitaxel treatment.

Rank	Genes	Log2 (FC)	Expression level	Validated target	Changes under pain condition
1	mmu-miR-181a-2-3p	3.25	Medium		
2	mmu-miR-323-5p	2.76	Medium		
3	mmu-miR-485-5p	2.55	High	Cdc42 and Rac1 (Zhang Z. et al., 2020); ASIC1 (Xu et al., 2020)	Nerve injury↓ (Zhang Z. et al., 2020); Inflammation pain↓ (Xu et al., 2020)
4	mmu-miR-191-3p	2.54	Medium		
5	mmu-let-7d-3p	2.09	High	Oprm1 (Li et al., 2021)	
6	mmu-let-7e-3p	2.02	Medium		
7	mmu-miR-138-1-3p	1.88	Medium		
8	mmu-miR-211-5p	1.87	Medium		
9	mmu-miR-615-3p	1.87	Medium		
10	mmu-miR-139-3p	1.70	Medium		
11	mmu-miR-369-5p	1.51	High		
1	mmu-miR-362-3p	–8.85	Medium		
2	mmu-miR-466b-3p	–7.60	Medium		Nerve injury↓ (Rau et al., 2010)
3	mmu-miR-466c-3p	–7.60	Medium		
4	mmu-miR-466p-3p	–7.60	Medium		
5	mmu-miR-107-3p	–6.43	Medium		
6	mmu-miR-19b-3p	–6.13	High	Potassium channels (Sakai et al., 2017)	Nerve injury↑ (Sakai et al., 2017)
7	mmu-miR-339-5p	–5.85	Medium		Acupuncture↓ (Wang et al., 2014)
8	mmu-miR-365-3p	–5.85	Medium		
9	mmu-miR-1249-3p	–5.74	Medium		Nerve injury↓ (Dai et al., 2019)
10	mmu-miR-674-3p	–5.71	Medium		
11	mmu-miR-376b-5p	–5.66	High		
12	mmu-miR-30e-5p	–5.47	High		
13	mmu-miR-106b-5p	–5.26	Medium		
14	mmu-miR-29c-3p	–5.26	High	PRKCI (Jia et al., 2018)	diabetic db/db mice↑ (Jia et al., 2018)
15	mmu-miR-377-3p	–5.26	Medium		
16	mmu-miR-22-5p	–5.24	High		
17	mmu-miR-142a-3p	–5.11	High		
18	mmu-miR-144-5p	–5.04	Medium		Nerve injury↓ (Rau et al., 2010; Zhang et al., 2011)
19	mmu-miR-17-5p	–5.02	Medium	Potassium channels (Sakai et al., 2017)	Nerve injury↑ (Sakai et al., 2017)
20	mmu-miR-29a-5p	–4.99	Medium		
21	mmu-miR-29a-3p	–4.94	High		
22	mmu-miR-33-5p	–4.93	High	GDNF (Zhang et al., 2019)	Bupivacaine (Bv)-induced neural apoptosis↑ (Zhang et al., 2019); diabetic peripheral neuropathy (Bali et al., 2021)

**TABLE 4:** Shared differentially expressed miRNAs among neuropathic pain models.

MiRNAs	MiRNA changes		
	PTX	DPN (Bali et al., 2021)	SNI (Dai et al., 2019)
miR-532-5p	↓	↓	↓
miR-132-5p	↑	↓	↑
miR-376b-3p	↓	↓	↑

PTX, paclitaxel; DPN, diabetic peripheral neuropathy; SNI, spared nerve injury.

### Differentially expressed circRNAs induced by treatment using paclitaxel

**FIGURE 3:**
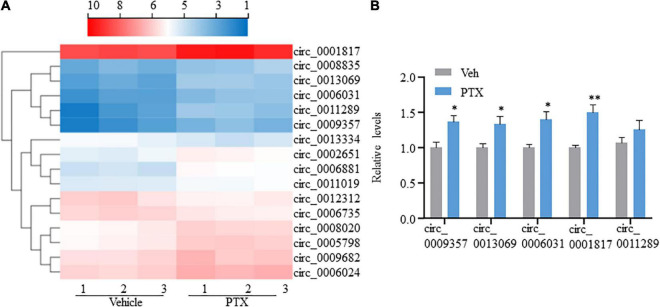
Transcriptome profiling and validation of circRNA expression in paclitaxel (PTX)-treated mice. **(A)** Hierarchical clustering analysis shows 16 differentially expressed circRNAs in vehicle and PTX groups. **(B)** Validation of differentially expressed circRNAs using quantitative polymerase chain reaction. Results are presented as mean ± SEM of 4–5 independent experiments. *P < 0.05; ^**^P < 0.01 compared with vehicle group by two-tailed unpaired *t*-test.

CircRNAs, a type of non-coding RNAs, have been identified as miRNA sponges that regulate the expression and function of coding mRNAs, and are involved in various biological processes including pain (Wang et al., 2018; Zhang et al., 2019; Lin et al., 2020; Song et al., 2020; Zhang H. H. et al., 2020). From our RNA-Seq data, 16 circRNAs were extracted as DEGs induced by treatment using PTX (Figure 3A and Table 5). RT-PCR was used to further validate several differentially expressed circRNAs showing high fold changes in expression or relative high expression levels. Our results show that the expression levels of mmu_circ_0009357, mmu_circ_0013069, mmu_circ_0006031, and mmu_circ_0001817 were increased by 1.37-, 1.33-, 1.40-, and 1.50-fold, respectively, by PTX treatment compared with their respective levels in control mice; these increases were statistically significant (*P* < 0.05) (Figure 3B). The expression level of mmu_circ_0011289 was also increased by 1.26-fold in response to PTX treatment compared with its levels in control mice, but this change did not reach statistical significance (*P* = 0.25) (Figure 3B).

**TABLE 5:** Differentially expressed circRNA induced by paclitaxel treatment.

Gene name	Gene symbol	Log2 (FC)	*Q*-value
mmu_circ_0013334	Dgki	–0.585	0.019
mmu_circ_0012312	Trrap	–0.485	0.043
mmu_circ_0006735	Tubb5	–0.413	0.030
mmu_circ_0009682	Stmn3	0.489	0.019
mmu_circ_0011019	Ssbp3	0.511	0.023
mmu_circ_0008020	Cox8a	0.541	0.002
mmu_circ_0006024	Arf3	0.609	<0.001
mmu_circ_0002651	Slc25a39	0.658	<0.001
mmu_circ_0005798	Grina	0.664	<0.001
mmu_circ_0001817	None	0.832	<0.001
mmu_circ_0008835	None	0.875	0.047
mmu_circ_0006881	Tubb4	1.012	<0.001
mmu_circ_0006031	Mll2	1.276	0.001
mmu_circ_0013069	Ptms	1.314	<0.001
mmu_circ_0009357	Prnp	1.443	0.007
mmu_circ_0011289	Arid1a	1.773	0.025

**FIGURE 4:**
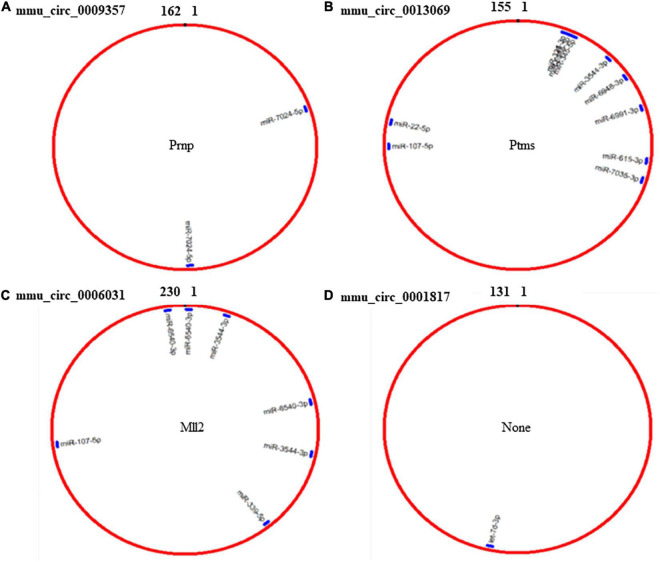
Prediction of miRNAs that interact with mmu_circ_0009357, mmu_circ_0013069, mmu_circ_0006031, and mmu_circ_0001817 by circMIR software. **(A)** Predicted binding site for miR-7024-5p on mmu_circ_0009357. **(B)** Combination site for 10 miRNAs on mmu_circ_0013069. **(C)** Predicted binding site for four miRNAs on mmu_circ_0006031. **(D)** Predicted binding site for let-7d-3p on mmu_circ_0001817.

Because circRNAs can function as miRNA sponges, thereby reducing miRNA ability to target mRNAs (Song et al., 2020), we used CircMIR software (BIOINF, The Affiliated Cancer Hospital of Nanjing Medical University, Nanjing, China) to predict miRNAs that interact with mmu_circ_0009357, mmu_circ_0013069, mmu_circ_0006031, and mmu_circ_0001817 (Figure 4). The predicted miRNAs, which were differentially expressed after induction using PTX, are shown in Figure 4 and Table 6.

**TABLE 6:** CircRNA and miRNA interaction predicted by CircMir software.

PTX-induced differentially expressed circRNAs	PTX-induced differentially expressed miRNAs
mmu_circ_0009357	mmu-miR-7024-5p
mmu_circ_0013069	mmu-miR-326-3p
	mmu-miR-6988-3p
	mmu-miR-345-5p
	mmu-miR-3544-3p
	mmu-miR-6948-3p
	mmu-miR-6991-3p
	mmu-miR-615-3p
	mmu-miR-7035-3p
	mmu-miR-107-5p
	mmu-miR-22-5p
mmu_circ_0006031	mmu-miR-6540-3p
	mmu-miR-3544-3p
	mmu-miR-339-5p
	mmu-miR-107-5p
mmu_circ_0001817	mmu-let-7d-3p

### Analysis of differentially expressed mRNAs

**FIGURE 5:**
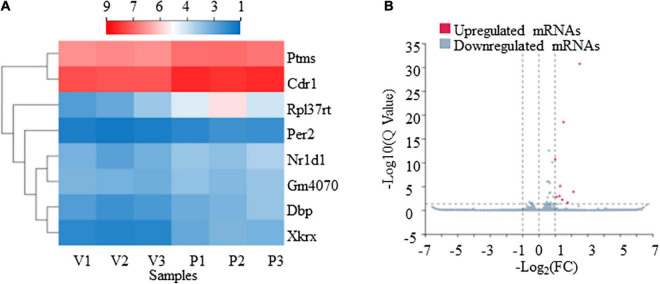
Transcriptome profiling of mRNAs in paclitaxel (PTX)-treated mice. **(A)** Hierarchical clustering analysis revealed eight differentially expressed mRNAs in vehicle and PTX groups. **(B)** Volcano plot of differentially expressed mRNAs following PTX administration.

From our RNA-Seq data, eight mRNAs and three lncRNAs were extracted as DEGs after treatment using PTX (Table 7). Most of these mRNAs showed minor changes in expression (Table 7). The eight differentially expressed mRNAs upregulated by treatment using PTX were as follows: encoding parathymosin (Ptms); encoding cerebellar degeneration related protein 1 (Cdr1); encoding ribosomal protein L37, retrotransposed (Rpl37rt); encoding period circadian regulator 2 (Per2); encoding nuclear receptor subfamily 1 group D member 1 (Nr1d1), also known as Gvin2; encoding GTPase, very large interferon inducible, family member 2 (Gm4070); encoding D-box binding PAR bZIP transcription factor (Dbp); and encoding XK related X-linked (Xkrx) (Table 7 and Figures 5A,B).

**TABLE 7:** Differentially expressed mRNA and lncRNA induced by paclitaxel treatment.

Gene ID	Gene name	Type	Log2 (FC)	*Q*-value
100042856	Gm4070	mRNA	0.467	0.034
69202	Ptms	mRNA	0.514	<0.001
631990	Cdr1	mRNA	0.627	<0.001
18627	Per2	mRNA	0.710	0.023
217166	Nr1d1	mRNA	0.911	0.001
13170	Dbp	mRNA	1.096	0.002
331524	Xkrx	mRNA	1.520	<0.001
100502825	Rpl37rt	mRNA	2.126	<0.001
BGIG10090_45213	BGIG10090_45213	lncRNA	–0.566	0.023
105246404	Gm41696	lncRNA	0.701	<0.001
BGIG10090_42549	BGIG10090_42549	lncRNA	2.515	<0.001

**FIGURE 6:**
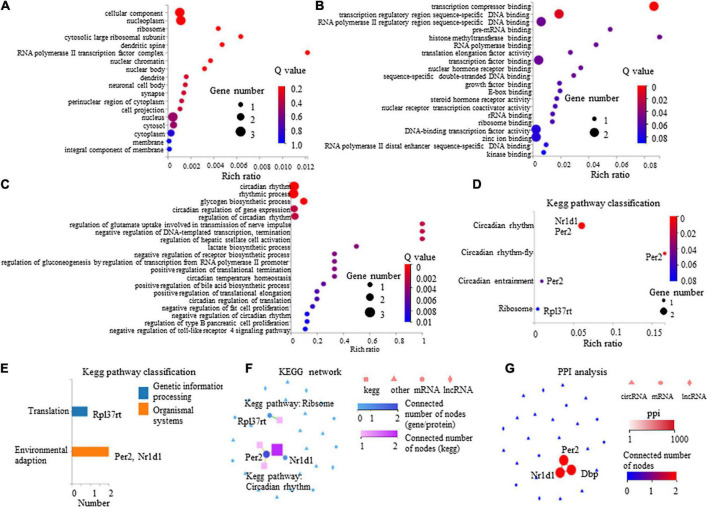
Gene Ontology (GO) enrichment analysis and Kyoto Encyclopedia of Genes and Genomes (KEGG) pathway of eight differentially expressed mRNAs in paclitaxel (PTX)-treated mice. **(A)** GO cellular component analysis. **(B)** GO molecular function analysis. **(C)** GO biological process analysis. **(D)** KEGG pathway enrichment analysis. **(E)** KEGG pathway classification analysis. **(F,G)** Connection between *Nr1d1*, *Per2*, and *Dbp* shown by KEGG network **(F)** and protein-protein interactions (PPI) analysis **(G)**.

The GO enrichment analysis indicated that these DEGs showed nucleus-, cytoplasm-, membrane-, and dendrite-related functions (Figure 6A), and were also associated with transcription-, translation-, and circadian rhythm-related signaling (Figures 6B,C).

The KEGG pathway analysis was conducted to evaluate the major pathways associated with these eight differentially expressed mRNAs showing upregulated expression. Our results indicate that among these eight mRNAs, Nr1d1 and Per2 were important in the regulation of circadian rhythm and were associated with environmental adaption (Figures 6D,E). Rpl37rt was enriched in ribosome and important in the regulation of translation (Figures 6D,E). KEGG network analysis showed that Nr1d1 and Per2 were connected to others, indicating that they may function jointly (Figure 6F). Protein-protein interactions (PPI) analysis also showed that Nr1d1, Per2, and Dbp shared a common network (Figure 6G).

Starobova et al. (2020) recently described changes in differential gene expression in the DRG following treatment using vincristine, oxaliplatin, or cisplatin. Therefore, we compared PTX-induced DEGs assessed in our present study with those induced by oxaliplatin or cisplatin (Starobova et al., 2020) (Supplementary Datasheet 4). Although five genes (*Alas2*, *Hbb-bt*, *Itgb2l*, *Ly6c2*, and *Nr4a3*) were shared among vincristine, oxaliplatin, and cisplatin (Starobova et al., 2020), none of these genes showed significant changes in expression following treatment with PTX. However, Dbp mRNA showed significant changes in expression levels following treatment using PTX or oxaliplatin (Supplementary Datasheet 4). Moreover, Cdr1 mRNA also showed significant changes in expression levels following treatment using PTX or cisplatin (Supplementary Datasheet 4). We then compared our results with those obtained in a previous study on cisplatin-induced gene expression changes in A/J mice (Lessans et al., 2019). Our results show that *Dbp* was also a shared gene induced by treatment using either PTX or cisplatin (Supplementary Datasheet 5). Megat et al. (2019) also sequenced input mRNA, equivalent to the DRG transcriptome, and translating ribosome affinity purification (TRAP) mRNAs associated with translating ribosomes in the Nav1.8 subset of DRG neurons (TRAP-seq) from naive mice and mice with treatment using PTX (at day 10 after treatment). Input transcriptome analysis revealed that only 4 genes were upregulated (Dpep2, Car3, Iah1, and Arhgef4) and 1 was downregulated (Plcb3) in the DRG by treatment using PTX (Megat et al., 2019). Yet, none of these genes were overlapped with the eight differentially expressed mRNAs in the current work.

We previously reported that nerve injury induces changes in thousands of coding and non-coding RNA in injured DRGs (Wu et al., 2016); therefore, in our present study, we compared DEGs in the DRGs induced using PTX with DEGs in the DRGs induced using nerve injury. Our results indicate that none of PTX-induced DEGs showed significant expression changes in the DRGs subjected to nerve injury (Supplementary Table 1).

## Discussion

In this study, we used RNA-Seq and in-depth gene expression analysis to explore gene expression profiles in the DRGs of PTX-treated mice. Among the identified 67,228 genes, 345 microRNAs, 6 mRNAs, 3 lncRNAs, and 16 circRNAs were differentially expressed in response to treatment using PTX. Several previous studies have reported on DEGs in different neuropathic pain models; therefore, we compared DEG expression and summarized some of genes shared in these neuropathic pain models. We also used RT-PCR to validate the changes in the expression levels of several miRNAs and circRNAs.

MiRNAs are important in regulating pain transmission, and are, therefore, increasingly investigated for their emerging therapeutic potential (Sakai et al., 2017; Dai et al., 2019; Bali et al., 2021). In our present study, we identified 253 differentially expressed miRNAs (70 upregulated and 183 downregulated miRNAs) in PTX-treated mice. Among these miRNAs, several have been shown to contribute to pain regulation (Rau et al., 2010; Zhang et al., 2011; Wang et al., 2014; Sakai et al., 2017; Jia et al., 2018; Dai et al., 2019; Zhang and Wang, 2019; Xu et al., 2020; Zhang Z. et al., 2020; Li et al., 2021). Sakai et al. (2017) reported that miR-17-92 cluster members, including miR-17, miR-18a, miR-19a, miR-19b, miR-20a, and miR-92a, are consistently upregulated in primary sensory neurons after nerve injury, and that the miR-17-92 cluster cooperatively regulates the function of multiple voltage-gated potassium channel subunits, thereby perpetuating mechanical allodynia. However, another study reported that these miRNAs show unchanged expression levels in rats with SNI-induced neuropathic pain (Dai et al., 2019). Curiously, in our present study, most of these miRNAs were downregulated by PTX treatment, as shown by our RNA-Seq data. We then compared PTX induced-differentially expressed miRNAs with DEGs reported in nerve injury and diabetes induced-neuropathic pain models (Dai et al., 2019; Bali et al., 2021). Our results indicate that only mmu-miR-532-5p showed consistently downregulated expression among these three models of neuropathic pain. The shared differentially expressed miRNAs may be important in pain pathogenesis and warrant further investigation. Although the data obtained using high-throughput screening RNA-Seq provide many crucial clues in the study of pain genesis, further validation studies are necessary to elucidate the mechanisms involved in chronic pain development.

CircRNAs, a type of non-coding RNAs, can mediate the functions of diverse molecules including non-coding RNAs, mRNAs, DNA, and proteins. Thus, circRNAs play critical roles in tumor genesis and development, in sensitivity to radiation and chemotherapy, and in pain (Wang et al., 2018; Kristensen et al., 2019; Zhang et al., 2019; Lin et al., 2020; Song et al., 2020; Zhang H. H. et al., 2020). In our present study, we found that 16 circRNAs in the DRG showed altered expression levels in response to treatment using PTX. However, no overlapping circRNAs were found in comparing these circRNAs with those dysregulated in mice with diabetes mellitus (Zhang H. H. et al., 2020). CircRNAs usually sponge microRNAs to regulate the expression and function of coding mRNA (Zhang et al., 2019). Our results also show that PTX-induced differentially expressed circRNAs interacted with one or several PTX-induced differentially expressed miRNAs. Among these miRNAs, miR-339 shows upregulated expression in acupuncture, indicating that miR-339 may be involved in acupuncture analgesia (Wang et al., 2014). The miRNA miR-15b plays a critical role in oxaliplatin-induced chronic neuropathic pain via downregulation of BACE1 expression (Ito et al., 2017). Therefore, it is interesting to find out whether a circRNA-miRNA regulatory pathway underlies PTX-induced neuropathic pain.

Chemotherapy-induced peripheral neuropathy and neuropathic pain can be induced by numerous anticancer agents, such as PTX, oxaliplatin, vincristine, and cisplatin, although these drugs have diverse mechanisms of action (Liang et al., 2020a; Starobova et al., 2020; Kang et al., 2021). PTX, which is a chemotherapeutic agent belonging to the taxane class, binds to cytoskeletal microtubules and enhances tubulin polymerization (Jordan and Wilson, 2004). Vincristine inhibits the assembly of β-tubulin (Gan et al., 2010). Platinum derivatives oxaliplatin and cisplatin bind to DNA and interfere with replication, transcription, and the cell cycle (Alcindor and Beauger, 2011; Dasari and Tchounwou, 2014). Several studies have reported on changes in differential gene expression in the DRG following treatment with PTX, vincristine, oxaliplatin, or cisplatin (Megat et al., 2019; Starobova et al., 2020; Kang et al., 2021). Megat et al. (2019) explored the changes of DRG transcriptome in the Nav1.8 subset of DRG neurons by PTX treatment and five differentially expressed genes were identified in the DRG by paclitaxel treatment. Sadly, none of them were overlapped with the PTX-induced differentially expressed mRNAs in the current study. In Dr. Megat’s work, they gathered all DRGs from cervical, thoracic, and lumbar levels while we precisely gathered lumbar DRGs. This might be one of the reasons which cause the difference. In another recent work by Sun et al. (2022), RNA-Seq research identified 384 differentially expressed mRNAs in the DRG of rats 14 days after PTX injection. As a full list of 384 DEGs is not available, it is not clear whether some mRNAs were overlapped in these studies. Nevertheless, the genes *Dbp* and *Cdr1* were shared between PTX and other chemotherapeutic agents by comparison of the results obtained in our present study with those obtained in other studies. *Dbp* encodes D site albumin promoter binding protein, which is a member of the Par bZIP transcription factor family; this protein can bind DNA as a homo- or heterodimer and is involved in the regulation of several circadian rhythm genes (Lopez-Molina et al., 1997). *Cdr1* encodes cerebellar degeneration related antigen 1. Further investigation is necessary to explore the role of these genes in PTX-induced neuropathic pain.

Our present study had several limitations. In this study, our RNA-Seq analysis revealed several differentially expressed mRNAs following treatment with PTX. However, previous studies have reported on expression changes in hundreds of genes in PTX-induced DRGs (Zhang et al., 2016; Li et al., 2017; Mao et al., 2019). Previously, we found that PTX induces downregulation in the expression of *K2p1.1* mRNA and protein in DRG neurons (Mao et al., 2019). Downregulation of *K2p1.1* mRNA in the DRG is also attributed to paclitaxel-induced increase in DNMT3a expression levels in the DRG (Mao et al., 2019). However, changes in the expression levels of these genes were not observed in our present study. This is probably due to the large differences among animals treated with PTX. We also observed some miRNAs are oppositely regulated under different pain conditions. The different causative origin may be one of the reasons. On the other hand, the expression level of one miRNA is probably different in the development and maintenance of neuropathic pain. Finally, although transcriptomic analysis of nervous tissue has greatly extended our understanding of pain mechanisms and progression, validation and mechanistic exploration of DEG expression are essential and warrant further investigation.

## Data availability statement

The data presented in the study are deposited in the BIG Sub repository (https://ngdc.cncb.ac.cn/gsa/), accession number CRA007673.

## Ethics statement

The animal study was reviewed and approved by Institutional Animal Ethics Committee of the Xi’an Jiaotong University Health Science Center.

## Author contributions

LL and QM initiated the project, designed the experiments, and analyzed the data. LT, JW, XQZ, HC, XuZ, XL, ZG, and XiZ did behavioral tests and molecular experiment. QM uploaded the RNA-Seq raw data to BIG Sub. LL wrote the manuscript. All authors read and approved the final manuscript.

## References

[B1] Alcindor T. Beauger N. (2011). Oxaliplatin: a review in the era of molecularly targeted therapy. *Curr. Oncol.* 18 18–25. 10.3747/co.v18i1.708 21331278 PMC3031353

[B2] Bali K. K. Gandla J. Rangel D. R. Castaldi L. Mouritzen P. Agarwal N. (2021). A genome-wide screen reveals microRNAs in peripheral sensory neurons driving painful diabetic neuropathy. *Pain* 162 1334–1351. 10.1097/j.pain.0000000000002159 33492037

[B3] Cock P. J. Fields C. J. Goto N. Heuer M. L. Rice P. M. (2010). The Sanger FASTQ file format for sequences with quality scores, and the Solexa/Illumina FASTQ variants. *Nucleic Acids Res.* 38 1767–1771. 10.1093/nar/gkp1137 20015970 PMC2847217

[B4] Dai D. Wang J. Jiang Y. Yuan L. Lu Y. Zhang A. (2019). Small RNA sequencing reveals microRNAs related to neuropathic pain in rats. *Braz. J. Med. Biol. Res.* 52:e8380. 10.1590/1414-431X20198380 31531524 PMC6753853

[B5] Dasari S. Tchounwou P. B. (2014). Cisplatin in cancer therapy: molecular mechanisms of action. *Eur. J. Pharmacol.* 740 364–378. 10.1016/j.ejphar.2014.07.025 25058905 PMC4146684

[B6] Finnerup N. B. Kuner R. Jensen T. S. (2021). Neuropathic pain: from mechanisms to treatment. *Physiol. Rev.* 101 259–301. 10.1152/physrev.00045.2019 32584191

[B7] Gan P. P. McCarroll J. A. Po’uha S. T. Kamath K. Jordan M. A. Kavallaris M. (2010). Microtubule dynamics, mitotic arrest, and apoptosis: drug-induced differential effects of betaIII-tubulin. *Mol. Cancer Ther.* 9 1339–1348. 10.1158/1535-7163.MCT-09-0679 20442307

[B8] Ito N. Sakai A. Miyake N. Maruyama M. Iwasaki H. Miyake K. (2017). miR-15b mediates oxaliplatin-induced chronic neuropathic pain through BACE1 down-regulation. *Br. J. Pharmacol.* 174 386–395. 10.1111/bph.13698 28012171 PMC5301044

[B9] Jia L. Wang L. Chopp M. Li C. Zhang Y. Szalad A. (2018). MiR-29c/PRKCI Regulates Axonal Growth of Dorsal Root Ganglia Neurons Under Hyperglycemia. *Mol. Neurobiol.* 55 851–858. 10.1007/s12035-016-0374-5 28070856 PMC5577385

[B10] Jordan M. A. Wilson L. (2004). Microtubules as a target for anticancer drugs. *Nat. Rev. Cancer* 4 253–265. 10.1038/nrc1317 15057285

[B11] Kang L. Tian Y. Xu S. Chen H. (2021). Oxaliplatin-induced peripheral neuropathy: clinical features, mechanisms, prevention and treatment. *J. Neurol.* 268 3269–3282. 10.1007/s00415-020-09942-w 32474658

[B12] Kristensen L. S. Andersen M. S. Stagsted L. V. W. Ebbesen K. K. Hansen T. B. Kjems J. (2019). The biogenesis, biology and characterization of circular RNAs. *Nat. Rev. Genet.* 20 675–691. 10.1038/s41576-019-0158-7 31395983

[B13] Kukurba K. R. Montgomery S. B. (2015). RNA sequencing and analysis. *Cold Spring Harb. Protoc.* 2015 951–969. 10.1101/pdb.top084970 25870306 PMC4863231

[B14] Langmead B. Salzberg S. L. (2012). Fast gapped-read alignment with Bowtie 2. *Nat. Methods* 9 357–359. 10.1038/nmeth.1923 22388286 PMC3322381

[B15] Lessans S. Lassiter C. B. Carozzi V. Heindel P. Semperboni S. Oggioni N. (2019). Global transcriptomic profile of dorsal root ganglion and physiological correlates of cisplatin-induced peripheral neuropathy. *Nurs. Res.* 68 145–155. 10.1097/NNR.0000000000000338 30586060

[B16] Li X. Chen Y. Wang J. Jiang C. Huang Y. (2021). Lung cancer cell-derived exosomal let-7d-5p down-regulates OPRM1 to promote cancer-induced bone pain. *Front. Cell Dev. Biol.* 9:666857. 10.3389/fcell.2021.666857 34124049 PMC8188355

[B17] Li Y. Tatsui C. E. Rhines L. D. North R. Y. Harrison D. S. Cassidy R. M. (2017). Dorsal root ganglion neurons become hyperexcitable and increase expression of voltage-gated T-type calcium channels (Cav3.2) in paclitaxel-induced peripheral neuropathy. *Pain* 158 417–429. 10.1097/j.pain.0000000000000774 27902567 PMC5303135

[B18] Liang L. Lutz B. M. Bekker A. Tao Y. X. (2015). Epigenetic regulation of chronic pain. *Epigenomics* 7 235–245. 10.2217/epi.14.75 25942533 PMC4422180

[B19] Liang L. Wei J. Tian L. Padma Nagendra B. V. Gao F. Zhang J. (2020a). Paclitaxel induces sex-biased behavioral deficits and changes in gene expression in mouse prefrontal cortex. *Neuroscience* 426 168–178. 10.1016/j.neuroscience.2019.11.031 31846751

[B20] Liang L. Wu S. Lin C. Chang Y. J. Tao Y. X. (2020b). Alternative splicing of Nrcam gene in dorsal root ganglion contributes to neuropathic pain. *J. Pain* 21 892–904. 10.1016/j.jpain.2019.12.004 31917219 PMC7380119

[B21] Liang L. Zhang J. Tian L. Wang S. Xu L. Wang Y. (2020c). AXL signaling in primary sensory neurons contributes to chronic compression of dorsal root ganglion-induced neuropathic pain in rats. *Mol. Pain* 16:1744806919900814. 10.1177/1744806919900814 31884887 PMC6970473

[B22] Lin J. Shi S. Chen Q. Pan Y. (2020). Differential expression and bioinformatic analysis of the circRNA expression in migraine patients. *Biomed. Res. Int.* 2020:4710780. 10.1155/2020/4710780 33178826 PMC7607275

[B23] Lopez-Molina L. Conquet F. Dubois-Dauphin M. Schibler U. (1997). The DBP gene is expressed according to a circadian rhythm in the suprachiasmatic nucleus and influences circadian behavior. *EMBO J.* 16 6762–6771. 10.1093/emboj/16.22.6762 9362490 PMC1170280

[B24] Mao Q. Wu S. Gu X. Du S. Mo K. Sun L. (2019). DNMT3a-triggered downregulation of K2p 1.1 gene in primary sensory neurons contributes to paclitaxel-induced neuropathic pain. *Int. J. Cancer* 145 2122–2134. 10.1002/ijc.32155 30684388 PMC6660426

[B25] Megat S. Ray P. R. Moy J. K. Lou T. F. Barragan-Iglesias P. Li Y. (2019). Nociceptor translational profiling reveals the ragulator-Rag GTPase complex as a critical generator of neuropathic pain. *J. Neurosci.* 39 393–411. 10.1523/JNEUROSCI.2661-18.2018 30459229 PMC6335757

[B26] Rau C. S. Jeng J. C. Jeng S. F. Lu T. H. Chen Y. C. Liliang P. C. (2010). Entrapment neuropathy results in different microRNA expression patterns from denervation injury in rats. *BMC Musculoskelet. Disord.* 11:181. 10.1186/1471-2474-11-181 20704709 PMC2927509

[B27] Sakai A. Saitow F. Maruyama M. Miyake N. Miyake K. Shimada T. (2017). MicroRNA cluster miR-17-92 regulates multiple functionally related voltage-gated potassium channels in chronic neuropathic pain. *Nat. Commun.* 8:16079. 10.1038/ncomms16079 28677679 PMC5504285

[B28] Scholz J. Finnerup N. B. Attal N. Aziz Q. Baron R. Bennett M. I. (2019). The IASP classification of chronic pain for ICD-11: chronic neuropathic pain. *Pain* 160 53–59. 10.1097/j.pain.0000000000001365 30586071 PMC6310153

[B29] Song G. Yang Z. Guo J. Zheng Y. Su X. Wang X. (2020). Interactions among lncRNAs/circRNAs, miRNAs, and mRNAs in neuropathic pain. *Neurotherapeutics* 17 917–931. 10.1007/s13311-020-00881-y 32632773 PMC7609633

[B30] Starobova H. Mueller A. Deuis J. R. Carter D. A. Vetter I. (2020). Inflammatory and neuropathic gene expression signatures of chemotherapy-induced neuropathy induced by vincristine, cisplatin, and oxaliplatin in C57BL/6J mice. *J. Pain* 21 182–194. 10.1016/j.jpain.2019.06.008 31260808

[B31] Sun W. Yang S. Wu S. Ba X. Xiong D. Xiao L. (2022). Transcriptome analysis reveals dysregulation of inflammatory and neuronal function in dorsal root ganglion of paclitaxel-induced peripheral neuropathy rats. *Mol. Pain* [Epub ahead of print]. 10.1177/17448069221106167 35610945 PMC10227877

[B32] Wang J. Y. Li H. Zhang L. Ma C. M. Wang J. L. Lai X. S. (2014). Adenosine as a probing tool for the mechanistic study of acupuncture treatment. *Clin. Exp. Pharmacol. Physiol.* 41 933–939. 10.1111/1440-1681.12304 25199539

[B33] Wang L. Feng Z. Wang X. Wang X. Zhang X. (2010). DEGseq: an R package for identifying differentially expressed genes from RNA-seq data. *Bioinformatics* 26 136–138. 10.1093/bioinformatics/btp612 19855105

[B34] Wang L. Luo T. Bao Z. Li Y. Bu W. (2018). Intrathecal circHIPK3 shRNA alleviates neuropathic pain in diabetic rats. *Biochem. Biophys. Res. Commun.* 505 644–650. 10.1016/j.bbrc.2018.09.158 30286957

[B35] Wu S. Marie Lutz B. Miao X. Liang L. Mo K. Chang Y. J. (2016). Dorsal root ganglion transcriptome analysis following peripheral nerve injury in mice. *Mol. Pain* 12:1744806916629048. 10.1177/1744806916629048 27030721 PMC4955972

[B36] Xu M. Wu R. Zhang L. Zhu H. Y. Xu G. Y. Qian W. (2020). Decreased MiR-485-5p contributes to inflammatory pain through post-transcriptional upregulation of ASIC1 in rat dorsal root ganglion. *J. Pain Res.* 13 3013–3022. 10.2147/JPR.S279902 33239909 PMC7682601

[B37] Zhang H. Li Y. de Carvalho-Barbosa M. Kavelaars A. Heijnen C. J. Albrecht P. J. (2016). Dorsal root ganglion infiltration by macrophages contributes to paclitaxel chemotherapy-induced peripheral neuropathy. *J. Pain* 17 775–786. 10.1016/j.jpain.2016.02.011 26979998 PMC4939513

[B38] Zhang H. Wang K. (2019). Downregulation of MicroRNA-33-5p protected bupivacaine-induced apoptosis in murine dorsal root ganglion neurons through GDNF. *Neurotox. Res.* 35 860–866. 10.1007/s12640-018-9994-z 30617464

[B39] Zhang H. H. Zhang Y. Wang X. Yang P. Zhang B. Y. Hu S. (2020). Circular RNA profile in diabetic peripheral neuropathy: analysis of coexpression networks of circular RNAs and mRNAs. *Epigenomics* 12 843–857. 10.2217/epi-2020-0011 32212929

[B40] Zhang H. Y. Zheng S. J. Zhao J. H. Zhao W. Zheng L. F. Zhao D. (2011). MicroRNAs 144, 145, and 214 are down-regulated in primary neurons responding to sciatic nerve transection. *Brain Res.* 1383 62–70. 10.1016/j.brainres.2011.01.067 21276775

[B41] Zhang S. B. Lin S. Y. Liu M. Liu C. C. Ding H. H. Sun Y. (2019). CircAnks1a in the spinal cord regulates hypersensitivity in a rodent model of neuropathic pain. *Nat. Commun.* 10:4119. 10.1038/s41467-019-12049-0 31511520 PMC6739334

[B42] Zhang Z. Li X. Li A. Wu G. (2020). miR-485-5p suppresses Schwann cell proliferation and myelination by targeting cdc42 and Rac1. *Exp. Cell Res.* 388:111803. 10.1016/j.yexcr.2019.111803 31877301

