# Supplementary material for: Transcriptome analysis of microRNAs, circRNAs, and mRNAs in the dorsal root ganglia of paclitaxel-induced mice with neuropathic pain
Source: Front Mol Neurosci. 2022 Aug 31;15:990260. doi: 10.3389/fnmol.2022.990260 (PMC9470859; doi:10.3389/fnmol.2022.990260)
Supplement: Supplementary file 1 [file Data_Sheet_1.ZIP › SUPPLEMENTARY TABLE 1.docx]

SUPPLEMENTARY TABLE 1. The expression changes of PTX-induced differentially expressed genes in nerve injured-dorsal root ganglions.

| Gene ID | Gene name | PTX treatment | | Nerve injury | |
| --- | --- | --- | --- | --- | --- |
|  |  | Log_2_(FC) | Q Value | Log_2_(FC) | Q Value |
| 100042856 | Gm4070 | 0.467 | 0.034 | 2.200 | 0.115 |
| 69202 | Ptms | 0.514 | <0.001 | -1.204 | 0.920 |
| 631990 | Cdr1 | 0.627 | <0.001 | - | - |
| 18627 | Per2 | 0.710 | 0.023 | -1.105 | 1 |
| 217166 | Nr1d1 | 0.911 | 0.001 | -1.402 | 0.302 |
| 13170 | Dbp | 1.096 | 0.002 | -1.217 | 0.782 |
| 331524 | Xkrx | 1.520 | <0.001 | - | - |
| 100502825 | Rpl37rt | 2.126 | <0.001 | - | - |
